# The maximum tumor growth rate predicts clinical outcomes of patients with small‐cell lung cancer undergoing first‐line chemotherapy plus immune‐checkpoint inhibitor therapy

**DOI:** 10.1002/cam4.5611

**Published:** 2023-01-11

**Authors:** Xiang Chen, Xueyuan Chen, Tingting Liu, Ting Zhou, Gang Chen, Huaqiang Zhou, Yan Huang, Wenfeng Fang, Yunpeng Yang, Ningning Zhou, Likun Chen, Silang Mo, Li Zhang, Yuanyuan Zhao

**Affiliations:** ^1^ Medical Oncology Department Sun Yat‐Sen University Cancer Center Guangzhou China; ^2^ State Key Laboratory of Oncology in South China Guangzhou China; ^3^ Collaborative Innovation Center for Cancer Medicine Guangzhou China

**Keywords:** chemoimmunotherapy, first‐line treatment, SCLC, small cell lung cancer, survival, tumor growth rate

## Abstract

**Background:**

Currently, no biomarkers can accurately predict survival outcomes in patients with SCLC undergoing treatment. Tumor growth rate (TGR; percent size change per month [%/m]) is suggested as an imaging predictor of response to anti‐cancer treatment. We aimed to evaluate the predictive role of the maximum TGR (TGRmax) for outcomes of small‐cell lung cancer (SCLC) patients undergoing first‐line chemotherapy plus immune‐checkpoint inhibitor (ICI) treatment.

**Methods:**

Patients with SCLC receiving first‐line chemotherapy plus immunotherapy were analyzed within this retrospective study. The X‐tile program was used to identify the cut‐off value of TGRmax based on maximum progression‐free survival (PFS) stratification. The Kaplan–Meier methods and Cox regression models were used to evaluate the effect of the presence of TGRmax on PFS and overall survival (OS).

**Results:**

In total, 104 patients were evaluated. Median (range) TGRmax was −33.9 (−65.2 to 21.6) %/m and the optimal cut‐off value of TGRmax was −34.3%/m. Multivariate Cox regression analysis revealed that patients with TGRmax > −34.3%/m was associated with shorter PFS (hazard ratio [HR], 2.81; 95% CI, 1.71–4.63; *p* < 0.001) and OS (HR, 3.17; 95% CI, 1.41–7.08; *p* = 0.005). In patients who received partial response (PR), Kaplan–Meier survival analyses showed that superior PFS and OS (*p* = 0.005 and *p* = 0.009, respectively) benefit was observed when TGRmax ≤−34.3%/m.

**Conclusions:**

SCLC patients with TGRmax > −34.3%/m had worse PFS and OS in first‐line ICI plus platin‐based chemotherapy. TGRmax could independently serve as an early biomarker to predict the benefit from chemoimmunotherapy.

## Graphical abstract

SCLC patients with TGRmax > −34.3%/m had worse PFS and OS in first‐line immunotherapy plus platin‐based chemotherapy.

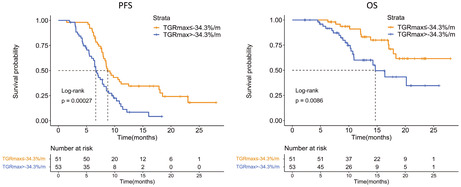

## 1 INTRODUCTION

Small‐cell lung cancer (SCLC) is a highly aggressive and fatal disease in which approximately two‐thirds of patients have metastatic disease when clinically diagnosed.^1^, ^2^ Platinum‐doublet chemotherapy has been the standard of care since 1980s and there has been minimal improvement in the therapeutic strategies and clinical outcomes over recent decades in patients with extensive‐stage SCLC (ES‐SCLC) until immunotherapy began to be applied.^3^, ^4^, ^5^ Compared with chemotherapy alone, the addition of the anti‐programmed cell death 1 ligand 1 (PD‐L1) antibodies significantly improved the overall survival (OS) of patients with ES‐SCLC both in the IMpower 133 and CASPIAN clinical trials.^6^, ^7^ Based on the results of these two studies, anti‐PD‐L1 antibodies were approved in the first‐line setting for patients with ES‐SCLC by the U.S. Food and Drug Administration.^8^ However, the Kaplan–Meier survival analyses‐based OS curves separated at around 8 months in the two studies. This suggested a discrepancy in efficacy at this time point, implying that only a subset of patients could benefit from the addition of immune‐checkpoint inhibitors (ICIs) to chemotherapy. Additionally, to the best of our knowledge, no biomarkers can accurately predict survival outcomes in patients with SCLC undergoing treatment to guide the use of ICIs.^9^

The tumor growth rate (TGR) was suggested as a feasible tool to evaluate therapeutic efficiency.^10^ The TGR is calculated by the change in tumor volume according to the Response Evaluation Criteria in Solid Tumors (RECIST)‐defined sum of the longest diameters of the target lesions over time between two CT imaging scans (%/month; %/m). Previous studies have proven that the TGR was independently associated with treatment response or prognosis in patients with neuroendocrine tumors, renal cell carcinoma, hepatocellular carcinoma, and prostate cancer.^11^, ^12^, ^13^, ^14^, ^15^, ^16^, ^17^ These findings suggested that the TGR formula, integrated with tumor growth kinetics, could reflect the change rate in tumor volume and might serve as an early radiological biomarker.

In this study, we sought to determine whether TGRmax, defined as the maximum TGR compared with baseline, would be an earlier and more robust measure of first‐line ICI plus platinum‐etoposide treatment response in extensive‐stage or relapsed SCLC. We explored whether TGRmax would provide a clinically meaningful signal of treatment benefit and if it could be used as a surrogate for progression‐free survival (PFS) or OS.

## 2 METHODS

### 2.1 Study population

An electronic medical record search was performed at Sun Yat‐Sen University Cancer Center (SYSUCC) between May 2018 and July 2021. Eligible patients with SCLC were adults who were previously untreated for extensive‐stage SCLC or those patients experiencing relapsed limited‐stage disease after initial therapy for more than 6 months. They should receive at least two cycles of ICI plus platin‐based chemotherapy in their first‐line therapy. Contrast‐enhanced computed tomography (CT) scans or positron emission tomography/computed tomography (PET/CT) examinations should be applied as imaging assessment at baseline (the latest radiological scan before first‐line treatment) and each follow‐up visit. The exclusion criteria were as follows: lack of an available radiological scan at baseline; lack of measurable target lesions by RECIST version 1.1 (RECIST 1.1) at baseline radiological evaluations,^18^ and lack of available follow‐up radiological scans. The study was approved by the Institutional Review Board of SYSUCC (ID: B2021‐030‐01). Written informed consent was waived because of the retrospective nature of the study.

For all included patients, we collected data including the patients' demographics: gender, age, smoking status, histology, clinical stage, Eastern Cooperative Oncology Group (ECOG) performance status (PS), treatment regimen, date of CT scans, the sum of the longest diameters of target lesions, location of tumor metastatic site at baseline, thoracic radiotherapy history, and baseline routine laboratory parameters (including lactate dehydrogenase (LDH)). Missing data were recorded as unavailable.

### 2.2 Assessments and endpoints

Assuming the tumor volume change follows an exponential law, we expressed the TGR as the percentage change in tumor volume per month using a published TGR calculation formula^10^: TGR = 100 × (exp (TG) − 1), TG = 3 × log (D2/D1)/t (months). Tumor size (D) was determined using the sum of the longest diameters of target lesions only (according to RECIST v1.1); non‐target and new lesions were not considered. D1 and D2 indicate tumor sizes at evaluation dates 1 and 2; and t (months) = (date 2 – date 1 + 1)/30.44, representing the time interval in months between two radiological imaging evaluations. The TGR was calculated each time when follow‐up radiological scans were carried out and TGRmax would be the one with maximum value among all the TGRs obtained during the whole course of first‐line treatment. A negative value indicates tumor shrinkage while a positive value indicates tumor growth.

We defined PFS as the time from baseline imaging evaluation to radiologically‐defined progression or death from any cause. OS was defined as the time from baseline imaging evaluation to death from any causes.

### 2.3 Statistical analysis

The X‐tile program (Yale University School of Medicine, New Haven, CT, USA) was employed to identify the optimal cut‐off value for TGRmax.^19^ Based on the TGRmax cut‐off value, patients were divided into two groups for further analysis. Categorical variables were expressed as numbers (%) and compared using the Chi‐square test or the Fisher's exact test. According to the normality of the data, an independent T‐test or Mann–Whitney U test were used for numerical variables, which were presented as median (range). Survival analysis was carried out using the Kaplan–Meier curves and the differences between the subgroups were compared using the log‐rank test. The relevance between patients' characteristics and survival prognosis was analyzed using univariate and multivariate Cox proportional hazards regression models. The concordance index (C‐index) and the area under the curve (AUC) were used to evaluate the predictive accuracy. A two‐sided *p* value of <0.05 would be considered statistically significant. All statistical analyses were conducted using the R software, version 4.1.0 (https://www.r‐project.org/).

## 3 RESULTS

### 3.1 Patient characteristics

Among 135 SCLC patients treated with first‐line ICI plus platinum‐etoposide, 104 individuals were eligible to be included in the analysis (Figure S1). In total, 93 patients were untreated extensive‐stage SCLC and 11 are relapsed limited‐stage disease after initial therapy for more than 6 months. The demographics of the included patients are shown in Table 1. Ninety patients (86.5%) were male and 69 (66.3%) were current or former smokers, with a median age 61 years (range, 19–76). Ninety six patients (88.3%) had a ECOG PS of 0–1, and only 19 (18.3%) ever received thoracic radiotherapy during their treatment. From the baseline imaging scans, 53 patients (51%) had more than three metastatic sites and 19 (18.3%) had brain metastasis. Fifty‐two (53.1%) were found to have an elevated LDH level (>250 U/L) among 98 patients who had baseline laboratory blood tests. For the best response based on RECIST 1.1, 87 (85.3%) patients achieved a partial response (PR), and 16 (15.4%) achieved stable disease (SD). The median time interval between the primary assessment and the start of first‐line treatment was 0.2 (0–1.0) months. The median PFS was 8.0 months (95% confidence interval [CI], 7.4–8.7 months). The median OS was not reached because only 33 people died in follow‐up (data maturity of 31.7%).

**TABLE 1:** Patient characteristics at baseline (*n* = 104)

Patient characteristics	No. (%)
Gender	
Male	90 (86.5)
Female	14 (13.5)
Age, years	
Median (range)	61 (19–76)
<61	49 (47.1)
≥61	55 (52.9)
Smoking status	
Never smoker	35 (33.7)
Current or former smoker	69 (66.3)
ECOG PS	
0–1	96 (92.3)
≥2	8 (7.7)
No. of metastatic sites	
≤3	51 (49.0)
>3	53 (51.0)
Brain metastasis	
No	83 (79.8)
Yes	21 (20.2)
Thoracic radiotherapy history	
No	85 (81.7)
Yes	19 (18.3)
LDH (*n* = 98)	
Normal	46 (46.9)
Elevated	52 (53.1)
Regime	
Platin‐based chemotherapy + Atezolizumab	33 (31.7)
Platin‐based chemotherapy + Durvalumab	51 (49.0)
Platin‐based chemotherapy + PD‐1 inhibitors^a^	20 (19.2)
TGRmax, %/m	
Median(range)	−33.9 (−65.2 to 21.6)
≤−34.3	51 (49.0)
>−34.3	53 (51.0)
RECIST response	
PR	87 (83.7)
SD	16 (15.4)
PD	1 (1.0)

Abbreviations: ECOG PS, Eastern Cooperative Oncology Group performance status; LDH, lactate dehydrogenase; PD, progressive disease; PD‐1, programmed cell death protein 1; PR, partial response; RECIST, Response Evaluation Criteria in Solid Tumors; SD, stable disease; TGRmax, the maximum tumor growth rate.

^a^ Sintilimab, Toripalimab, Tislelizumab, Camrelizumab, Nivolumab.

### 3.2 Cut‐off points of TGRmax

The Median TGRmax was −33.9 (−65.2 to 21.6) %/m. The X‐tile program was used to maximize the prognostic effects in predicting PFS and the optimal cut‐off point of TGRmax was set as −34.3%/m. Based on the TGRmax cut‐off point, patients were divided into two groups: TGRmax ≤ −34.3%/m (*n* = 51) and TGRmax > −34.3%/m (*n* = 53). The group ≤ −34.3%/m ranges from −65.1 to −34.3%/m and the other group ranges from −34.3 to 21.6%/m. The clinicopathological characteristics of the TGRmax strata are shown in Table 2. TGRmax ≤ −34.3%/m was significantly associated with RECIST‐defined best response (*p* < 0.001). There was no significant association between TGRmax and other factors, including gender, age, smoking history, ECOG PS, number of metastatic sites, brain metastasis, thoracic radiotherapy history, LDH level, and regime (all *p* > 0.05). Notably, 93 patients (89.4%) achieved TGRmax in their first follow‐up and the rest (10.6%) achieved TGRmax in second or later follow‐up. Ninety‐eight (94.2%) patients received their first follow‐up response evaluation after two cycles of therapy. Among patients who received radiological scans every two cycles of treatment (*n* = 98), as recommended by the National Comprehensive Cancer Network guideline (NCCN),^20^ 88 (89.8%) achieved TGRmax after receiving the first two cycles of therapy, which was about 6 weeks after drug administration.

**TABLE 2:** Association of TGRmax with other parameters (*n* = 104)

	TGRmax ≤−34.3%/m	TGRmax >−34.3%/m	*p* value
	(*n* = 51) No. (%)	(*n* = 53) No. (%)	
Gender			0.284
Male	46 (90.2)	44 (83.0)	
Female	5 (9.8)	9 (17.0)	
Age, years			0.512
Median (range)	61 (44–75)	61 (19–76)	
Smoking status			0.629
Never smoker	16 (31.4)	19 (35.8)	
Current or former smoker	35 (68.6)	34 (64.2)	
ECOG PS			0.955
0–1	47 (92.2)	49 (92.5)	
≥2	4 (7.8)	4 (7.5)	
No. of metastatic sites			0.698
≤3	26 (51.0)	25 (47.2)	
>3	25 (49.0)	28 (52.8)	
Brain metastasis			0.732
No	40 (78.4)	43 (81.1)	
Yes	11 (21.6)	10 (18.9)	
Thoracic radiotherapy history			0.729
No	41 (80.4)	44 (83.0)	
Yes	10 (19.6)	9 (17.0)	
LDH (*n* = 98)			0.685
Normal	24 (49.0)	22 (44.9)	
Elevated	25 (51.0)	27 (55.1)	
Regime			0.122
Platin‐based chemotherapy + Atezolizumab	21 (41.2)	12 (22.6)	
Platin‐based chemotherapy + Durvalumab	21 (41.2)	30 (56.6)	
Platin‐based chemotherapy + PD‐1 inhibitors^a^	9 (17.6)	11 (20.8)	
RECIST response			<0.001
PR	51 (100.0)	36 (67.9)	
SD	0 (0.0)	16 (30.2)	
PD	0 (0.0)	1 (1.9)	

Abbreviations: ECOG PS, Eastern Cooperative Oncology Group performance status; LDH, lactate dehydrogenase; PD, progressive disease; PD‐1, programmed cell death protein 1; PR, partial response; RECIST, Response Evaluation Criteria in Solid Tumors; SD, stable disease; TGRmax, the maximum tumor growth rate.

^a^ Sintilimab, Toripalimab, Tislelizumab, Camrelizumab, Nivolumab.

### 3.3 Association of TGRmax with clinical outcomes

**FIGURE 1:**
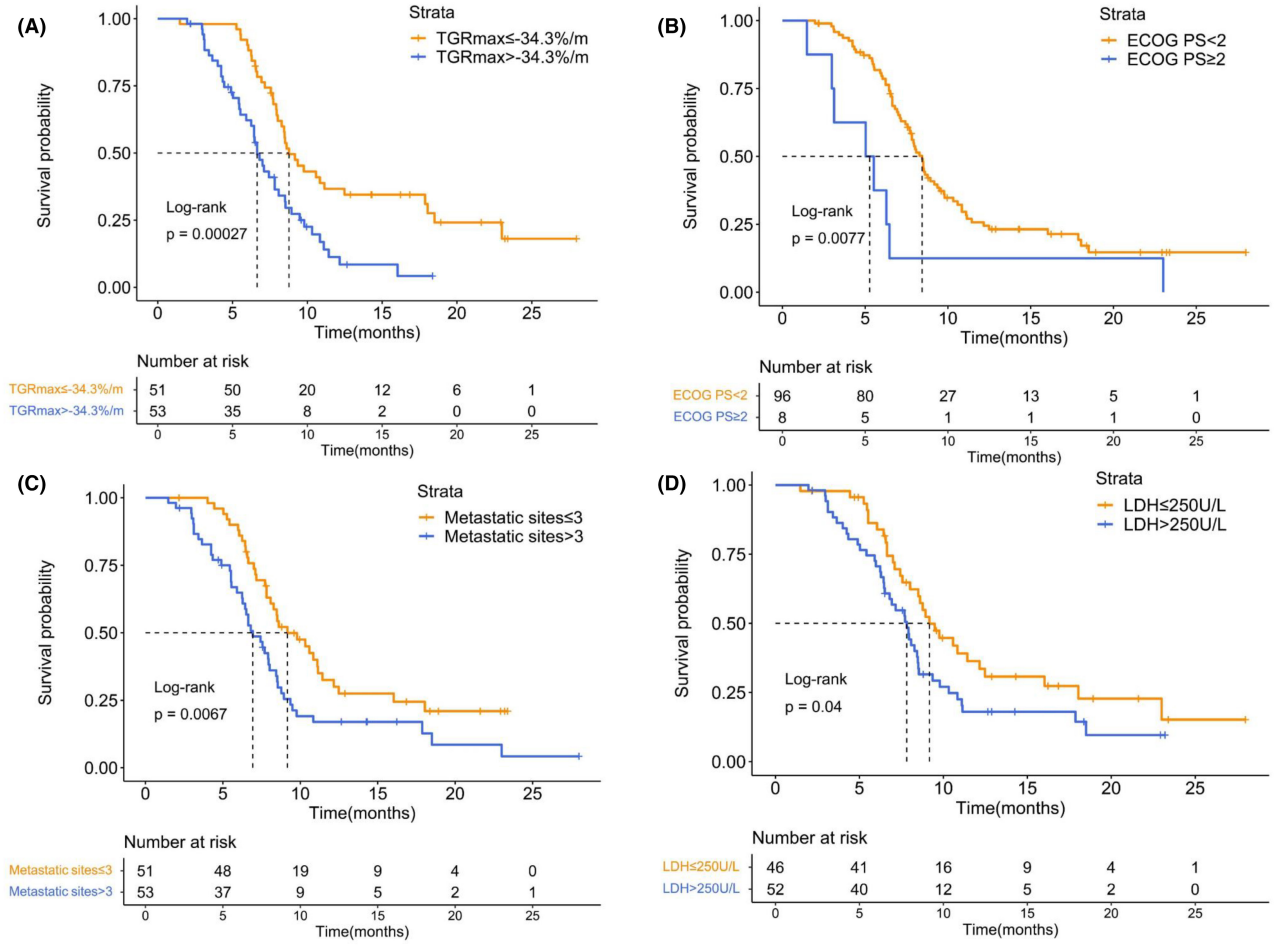
Kaplan–Meier analysis of PFS. (A) PFS by TGRmax. (B) PFS by ECOG PS. (C) PFS by number of metastatic sites. (D) PFS by LDH level. ECOG PS indicates Eastern Cooperative Oncology Group performance status; LDH, lactate dehydrogenase; PFS, progression‐free survival; TGRmax, the maximum tumor growth rate.

A median PFS benefit was observed in SCLC patients with TGRmax ≤ −34.3%/m, compared with those with TGRmax > −34.3%/m (median [95% CI], 8.8 [7.4–10.2] vs. 6.6 [5.9–7.4] months; *p* < 0.001) in the Kaplan–Meier survival analyses when receiving first‐line ICI plus platinum‐etoposide therapy (Figure 1). Besides, patients with an ECOG PS of 0–1 (*p* = 0.008), no more than three metastatic sites (*p* = 0.007) or a normal LDH level at baseline (*p* = 0.040) showed a longer PFS.

Univariate analyses revealed that the following factors were significantly associated with inferior PFS: TGRmax > −34.3%/m (hazard ratio [HR] 2.29; 95% CI, 1.45–3.63; *p* < 0.001), ECOG PS of two or more (HR 2.67; 95% CI, 1.26–5.62; *p* = 0.010), metastatic sites more than three (HR 1.84; 95% CI, 1.17–2.87; *p* = 0.008), and elevated LDH level (HR 1.62; 95% CI, 1.02–2.59; *p* = 0.042) (Table 3). The factors that were statistically significant in the univariate analyses were then included in the multivariate Cox model. We found that TGRmax > −34.3%/m (HR 2.81; 95% CI, 1.71–4.63; *p* < 0.001) remained significantly associated with shorter PFS (Table 4). Other independent variables associated with worse PFS included ECOG PS of two or more (HR 3.02; 95% CI, 1.38–6.61; *p* = 0.006), more than three metastatic sites (HR 1.91; 95% CI, 1.18–3.09; *p* = 0.009), and elevated LDH level (HR 1.65; 95% CI, 1.03–2.64; *p* = 0.039).

**TABLE 3:** Univariate analyses of progression‐free survival and overall survival

	PFS		OS	
	HR (95% CI)	*p*‐value	HR (95% CI)	*p*‐value
Gender				
Male	1 [Reference]	NA	1 [Reference]	NA
Female	1.15 (0.59–2.24)	0.681	1.12 (0.39–3.20)	0.832
Age, years				
<61	1 [Reference]	NA	1 [Reference]	NA
≥61	0.98 (0.63–1.52)	0.912	1.07 (0.53–2.16)	0.844
Smoking status				
Never smoker	1 [Reference]	NA	1 [Reference]	NA
Current or former smoker	0.89 (0.56–1.43)	0.640	0.72 (0.35–1.48)	0.368
ECOG PS				
0–1	1 [Reference]	NA	1 [Reference]	NA
≥2	2.67 (1.26–5.62)	0.010	3.26 (1.25–8.48)	0.016
No. of metastatic sites				
≤3	1 [Reference]	NA	1 [Reference]	NA
>3	1.84 (1.17–2.87)	0.008	2.22 (1.08–4.55)	0.030
Brain metastasis				
No	1 [Reference]	NA	1 [Reference]	NA
Yes	1.22 (0.71–2.12)	0.471	1.42 (0.61–3.28)	0.417
Thoracic radiotherapy history				
No	1 [Reference]	NA	1 [Reference]	NA
Yes	0.92 (0.52–1.62)	0.768	0.60 (0.23–1.56)	0.292
LDH				
Normal	1 [Reference]	NA	1 [Reference]	NA
Elevated	1.62 (1.02–2.59)	0.042	3.29 (1.45–7.43)	0.004
TGRmax, %/m				
≤−34.3%/m	1 [Reference]	NA	1 [Reference]	NA
>−34.3%/m	2.29 (1.45–3.63)	<0.001	2.55 (1.24–5.25)	0.011

Abbreviations: CI, confidence interval; ECOG PS, Eastern Cooperative Oncology Group performance status; HR, hazard ratio; LDH, lactate dehydrogenase; OS, overall survival; PFS, progression‐free survival; TGRmax, the maximum tumor growth rate.

**TABLE 4:** Multivariate analyses of progression‐free survival and overall survival

	PFS		OS	
	HR (95% CI)	*p*‐value	HR (95% CI)	*p*‐value
ECOG PS				
0–1	1 [Reference]	NA	1 [Reference]	NA
≥2	3.02 (1.38–6.61)	0.006	3.84 (1.43–10.35)	0.008
No. of metastatic sites				
≤3	1 [Reference]	NA	1 [Reference]	NA
>3	1.91 (1.18–3.09)	0.009	2.52 (1.15–5.53)	0.022
LDH				
Normal	1 [Reference]	NA	1 [Reference]	NA
Elevated	1.65 (1.03–2.64)	0.039	3.02 (1.31–6.98)	0.010
TGRmax, %/m				
≤−34.3%/m	1 [Reference]	NA	1 [Reference]	NA
>−34.3%/m	2.81 (1.71–4.63)	<0.001	3.17 (1.41–7.08)	0.005

Abbreviations: CI, confidence interval; ECOG PS, Eastern Cooperative Oncology Group performance status; HR, hazard ratio; LDH, lactate dehydrogenase; OS, overall survival; PFS, progression‐free survival; TGRmax, the maximum tumor growth rate.

**FIGURE 2:**
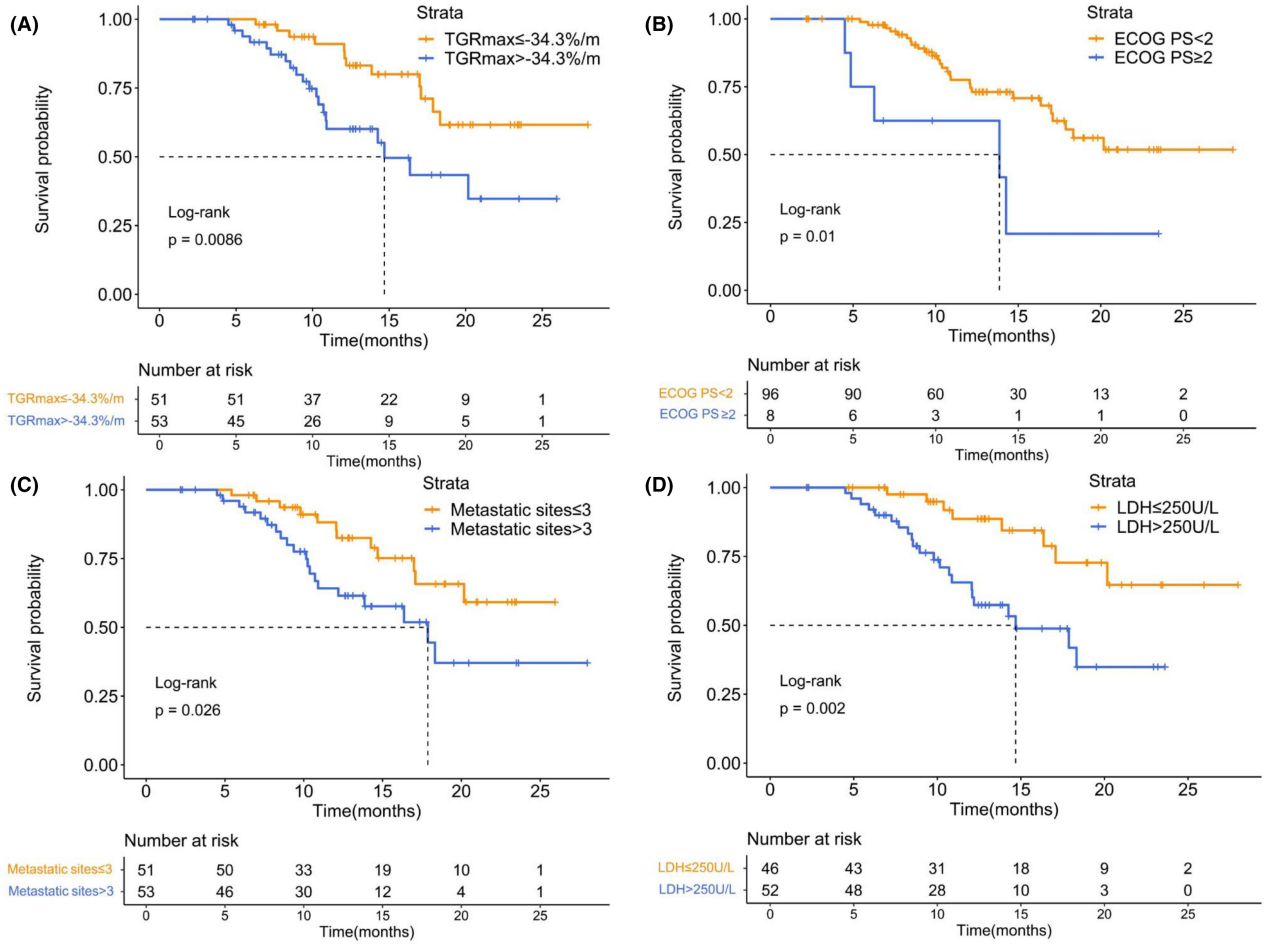
Kaplan–Meier analysis of OS. (A) OS by maximum tumor growth rate. (B) OS by ECOG PS. (C) OS by number of metastatic sites. (D) OS by LDH level. ECOG PS indicates Eastern Cooperative Oncology Group performance status; LDH, lactate dehydrogenase; OS, overall survival; TGRmax, the maximum tumor growth rate.

Similarly, Patients with TGRmax > −34.3%/m tended to have shorter OS than those with TGRmax ≤ −34.3%/m (median [95% CI], 14.7 [11.4–18.0] vs. not reached (NR) months; *p* = 0.009) (Figure 2). Moreover, patients with ECOG PS of two or more (*p* = 0.010), more than three metastatic sites (*p* = 0.026) or elevated LDH level (*p* = 0.002) exhibited a poorer OS.

Univariate Cox regression analyses of OS found that −34.3%/m TGRmax (*p* = 0.011), ECOG PS (*p* = 0.016), number of metastatic sites (*p* = 0.030), and LDH level (*p* = 0.004) were significant predictors (Table 3), and multivariate analyses also proved −34.3%/m TGR was a valid independent predictive factor of OS (HR 3.17; 95% CI, 1.41–7.08; *p* = 0.005) (Table 4).

**FIGURE 3:**
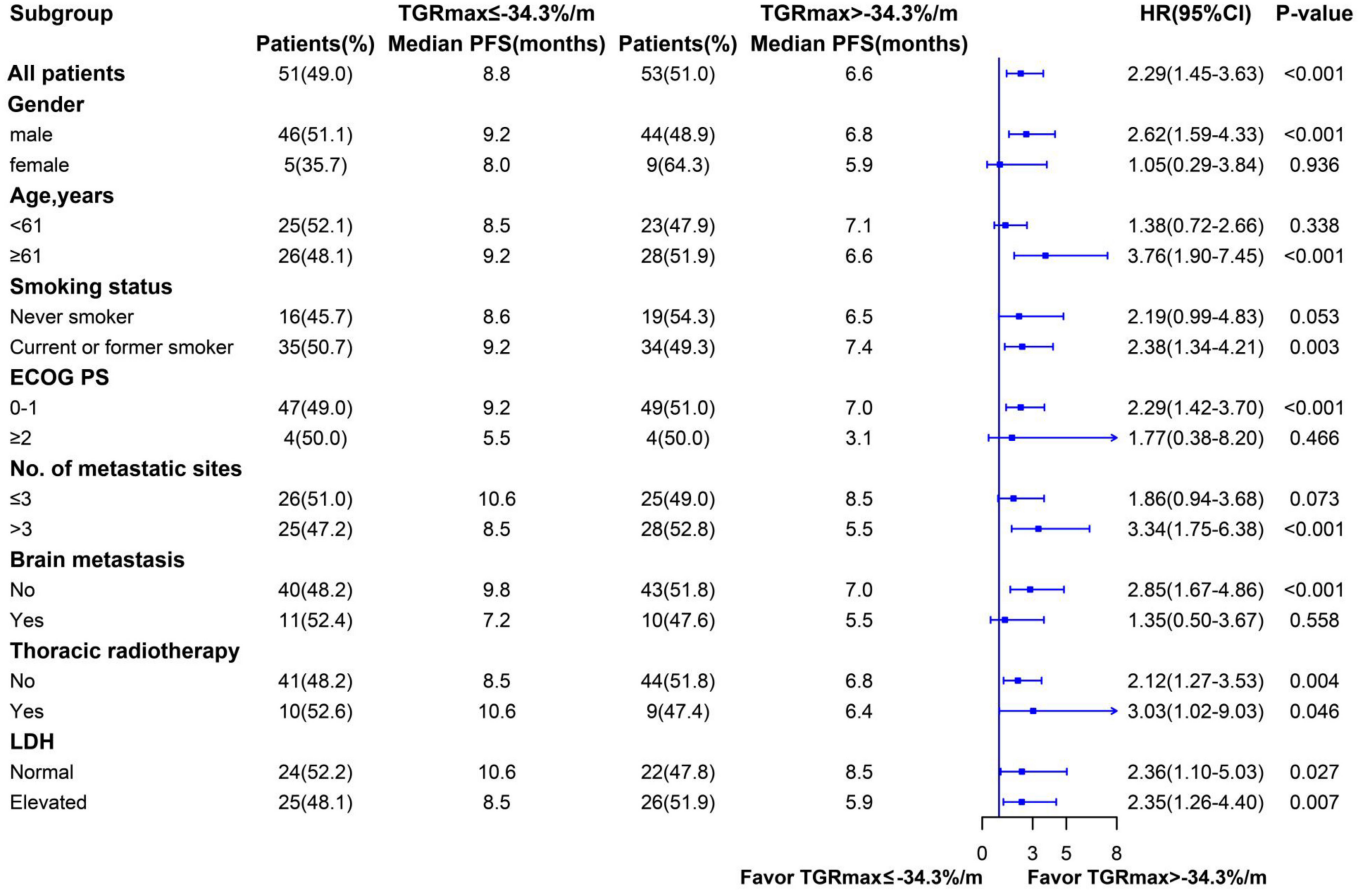
Subgroup analysis of PFS according to TGRmax stratification. Findings were examined by Cox proportional hazard regression analysis. CI indicates confidence interval; ECOG PS, Eastern Cooperative Oncology Group performance status; HR, hazard ratio; LDH, lactate dehydrogenase; PFS, progression‐free survival; TGRmax, the maximum tumor growth rate.

A subgroup analysis based on prognostic factors was conducted to further validate the effect of TGRmax on PFS. TGRmax played a significant predictable role in patients with SCLC among the subgroups including male, age over 61, current or former smoker, more than three metastatic sites, no brain metastasis, and a negative history of thoracic radiotherapy (all *p* < 0.01) (Figure 3). Patients with TGRmax > −34.3%/m in these specific groups were much more likely to have poorer survival outcomes. In the LDH level subgroup, LDH normal group (*p* = 0.027) and elevated group (*p* = 0.007) both show TGRmax > −34.3%/m was related to shorter PFS.

### 3.4 TGRmax in patients with RECIST‐defined PR

**FIGURE 4:**
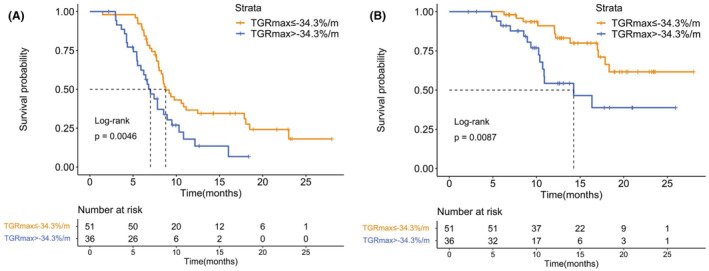
Kaplan–Meier analysis of (A) PFS and (B) OS by TGRmax in patients who achieved partial response after two cycles of treatment. OS indicates overall survival; PFS, progression‐free survival; TGRmax, the maximum tumor growth rate.

Most patients received a PR in our study and previous study^6^, ^7^; therefore, we carried out further exploration to validate the predictive value of TGRmax in patients with PR and the effectiveness of the cut‐off value set by X‐tile program. Patients who achieved a PR as their best response in the first‐line treatment according to the RECIST 1.1 criteria (*n* = 87) were stratified into two groups based on the TGRmax cut‐off value of −34.3%/m. Kaplan–Meier survival analyses showed that both superior PFS and OS (*p* = 0.005 and *p* = 0.009, respectively) benefit was observed when TGRmax ≤ −34.3%/m (*n* = 51) compared with those which did not (*n* = 27) in PR groups (Figure 4).

### 3.5 Predictive effect of TGRmax

**FIGURE 5:**
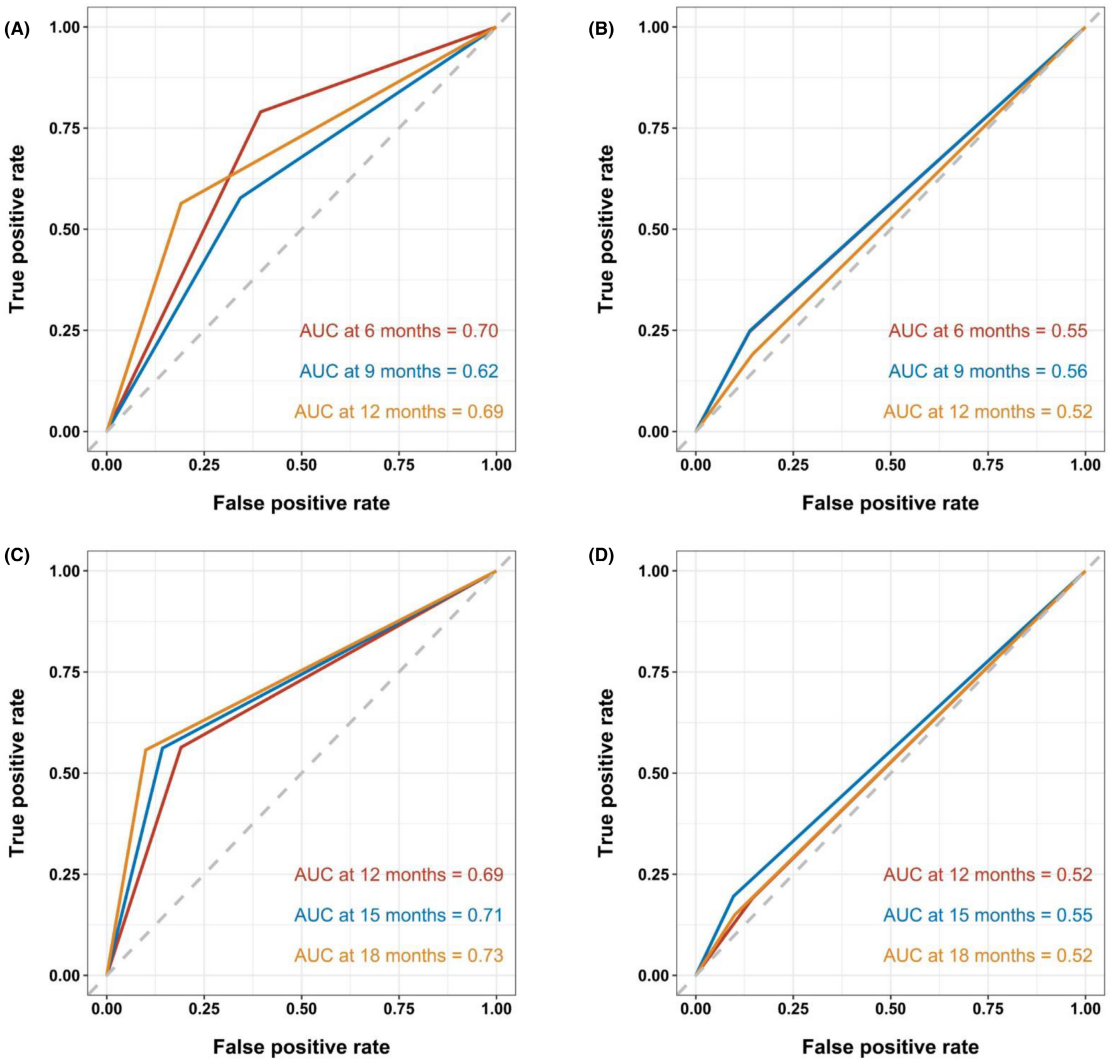
The AUCs of the probability of PFS at 6, 9, and 12 months by (A) TGRmax and (B) RECIST criteria, respectively and OS at 12, 15, and 18 months by (C) TGRmax and (D) RECIST criteria, respectively. AUC indicates area under the curve; RECIST, Response Evaluation Criteria in Solid Tumors; OS, overall survival; PFS, progression‐free survival; TGRmax, the maximum tumor growth rate.

The concordance between the clinical outcome parameters PFS or OS and TGRmax versus RECIST‐defined best response was then assessed. For PFS, the AUCs at 6, 9, and 12 months were 0.70, 0.62, and 0.69 for TGRmax while they were 0.63, 0.53 and 0.60 for response by the RECIST criteria (Figure 5). For OS, the AUCs at 12, 15, and 18 months were 0.69, 0.71, and 0.73 for TGRmax while they were 0.56, 0.53, and 0.55 for RECIST criteria. For PFS, the C‐index was 0.614 (95% CI, 0.560–0.668) and 0.555 (95% CI, 0.509–0.602) for TGRmax and RECIST criteria, respectively. For OS, the C‐index was 0.632 (95% CI, 0.549–0.716) and 0.529 (95% CI, 0.452–0.606) for TGRmax and RECIST criteria, respectively.

## 4 DISCUSSION

In the present study, we applied the tumor growth rate (TGR) to predict the survival outcomes in patients with extent‐stage or relapsed SCLC receiving a combination of chemotherapy plus immune checkpoint inhibitors in the first‐line setting. The results confirmed that TGRmax was independently associated with both PFS and OS of patients with extent‐stage or relapsed SCLC undergoing combined treatment.

The TGR has been proven to be associated with the survival outcomes of neuroendocrine tumors in previous studies. In the GREPONET Study, which included 222 patients with advanced grade 1/2 neuroendocrine tumors from the pancreas or small bowel, the TGR after 3 months of starting treatment or follow‐up without treatment was found to be an early radiological biomarker to predict PFS and had less variability than RECIST at 3 months.^16^ Besides, another posthoc analysis in grade 1/2 pancreatic and intestinal neuroendocrine tumors receiving lanreotide identified a pre‐treatment TGR cut‐off value of 4%/m. The threshold 4%/m was predictive for PFS and patients with pre‐treatment TGR >4%/m had a much higher risk of progression compared with those with pre‐treatment TGR ≤4%/m.^17^ In our analyses, we confirmed that TGRmax has the potential to predict the PFS and OS in patients with extent‐stage or relapsed SCLC, which is a type of neuroendocrine carcinoma. The results agreed with previous reports of the utilities of TGR in other neuroendocrine tumors. More recently, a retrospective study was carried out in 80 patients with advanced non‐small cell lung cancer (NSCLC) treated with ICI therapy without restriction to the patient's treatment line.^21^ That study showed that pre‐treatment TGR remained predictive when patients received immunotherapy for NSCLC. Our study was conducted with a view to evaluating the predictive effect of TGR on SCLC first‐line ICIs with chemotherapy. We used TGRmax to assess the treatment response and identified TGRmax cut‐off value of −34.3%/m. Based on our findings, more frequent or regular radiographic follow‐up interval would be necessary for patients with TGRmax >−34.3%/m, because this group is associated with a poorer clinical outcome. We believe that the predictive role of TGRmax is meaningful in clinical practice, since it could impact on the patients' efficacy evaluation plan and set the stage for early detection of disease progression.

SCLC is highly sensitive to the combination of chemotherapy and immunotherapy in the first‐line setting and about 70% patients will receive objective response according to the RECIST 1.1 criteria.^22^, ^23^ High as the response rate is, disease progression occurs in most patients in a short time. Thus, it is possible that heterogeneity exists among patients with partial response and further stratification would be necessary. To further explore the predictive value of TGRmax in the PR group, patients with PR in the treatment were extracted from the analysis, and we found the TGRmax cut‐off value of −34.3%/m could still play a predictive role in this group. Our work suggested that TGR measures subtle changes in tumor volume that the RECIST criteria is unable to identify, thus providing more sensitive and accurate information for clinicians. It would be helpful to implement TGR for further risk stratification in patients with SCLC who received PR during the treatment. Besides, in the predictive accuracy analysis, the AUC and C‐index analyses exhibited a better concordance for both PFS and OS as the clinical outcomes by TGRmax than by the RECIST‐defined response. Above all, the heterogeneity in survival benefit in SCLC first‐line treatment makes it necessary to further explore the risk level in patients who are classified as response to therapy according to the RECIST criteria. The TGR would be of value for use in combination with RECIST criteria in first‐line treatment strategies in SCLC, providing clinicians with additional useful information in patients achieved PR.

In further analysis, we noticed that in patients who received radiological scans every two cycles of treatment (*n* = 98), 88 (89.8%) achieved TGRmax after receiving the first two cycles of therapy. On the one hand, this suggests that the TGR could serve as an early radiological biomarker in real clinical practice. In addition, a CT examination after the first two cycles of treatment, that is, about 6 weeks after drug administration, would be of prime importance in SCLC first‐line treatment. Clinicians might need to be vigilant when patients achieved a TGR >−34.3%/m after the two cycles of treatment since chances are slim for the patients to achieve a more significant change in tumor volume in later treatments. On the other hand, our results suggested that most advanced SCLC tumors shrink significantly in the period up to first follow‐up, especially in the first two cycles of treatment, achieving the maximum volume change rate. The significant association between the maximum tumor shrinkage and survival in SCLC gives a signal that first‐line management strategies are vital in the totality of SCLC anti‐tumor treatment. The most effective therapeutic regimen would better be applied in the first‐line setting and maximizing early volume shrinkage will result in an increased potential of a better survival benefit.

There are several limitations to this study. First, the recognized predictors of immunotherapy, PD‐L1 status and the tumor mutational burden (TMB), were not included in our analysis. Although SCLC is characterized by a high TMB, PD‐L1 expression in SCLC is generally low or even absent.^24^, ^25^, ^26^, ^27^, ^28^ Their predictive value in SCLC is doubtful and most studies suggest that neither PD‐L1 expression nor the TMB predicts a clear benefit with ICIs in SCLC.^28^, ^29^, ^30^, ^31^ Second, due to the moderate sample size recruited from our single institute, statistical power might be limited. Further prospective or external studies in another cohort with a larger sample size is required to validate our conclusions. Third, because of the need for lesion measurement, patients who lacked an available CT scan at baseline or follow‐up visit or had no measurable target lesions would be excluded, which might have caused potential selection bias. Despite these limitations, our findings suggest that the role of ICIs plus chemotherapy in tumors with TGRmax ≤ −34.3%/m further improve the discrimination of the risk category. Appling TGRmax in clinical practice will contribute to better assessment the patient's risk of drug tolerance and improve follow‐up arrangements, thereby discerning early disease progression in patients who will probably have limited benefit from chemoimmunotherapy.

## 5 CONCLUSIONS

TGRmax was significantly associated with PFS and OS in patients with extensive‐stage or relapsed SCLC undergoing first‐line ICI plus platinum‐etoposide. TGRmax could independently serve as an early and effective biomarker to distinguish between patients who are more likely to benefit from chemoimmunotherapy and those who are not. Further larger prospective studies are needed to validate the predictive value of TGRmax and incorporate it into clinical application.

## AUTHOR CONTRIBUTIONS

**Xiang Chen:** Data curation (equal); formal analysis (lead); investigation (lead); writing – original draft (lead). **Xueyuan Chen:** Data curation (equal); software (equal); visualization (equal). **Tingting Liu:** Data curation (equal); software (equal); visualization (equal). **Ting Zhou:** Data curation (equal); resources (equal). **Gang Chen:** Data curation (equal); resources (equal). **Huaqiang Zhou:** Writing – review and editing (equal). **Yan Huang:** Data curation (equal); resources (equal). **Wen F Fang:** Data curation (equal); resources (equal). **Yun P Yang:** Data curation (equal); resources (equal). **Ningning Zhou:** Data curation (equal); resources (equal). **Likun Chen:** Data curation (equal); resources (equal). **Silang Mo:** Software (equal); validation (lead). **Li Zhang:** Conceptualization (equal); methodology (equal); project administration (equal); supervision (lead).

## FUNDING INFORMATION

None declared.

## CONFLICT OF INTEREST

All authors have no conflicts of interest to declare.

## Supporting information


Figure S1.
