# Supplementary material for: The maximum tumor growth rate predicts clinical outcomes of patients with small‐cell lung cancer undergoing first‐line chemotherapy plus immune‐checkpoint inhibitor therapy
Source: Cancer Med. 2023 Jan 11;12(7):8122–33. doi: 10.1002/cam4.5611 (PMC10134330; doi:10.1002/cam4.5611)
Supplement: Supplementary file 1 — Figure S1. [file CAM4-12-8122-s001.pptx]

## Slide 1
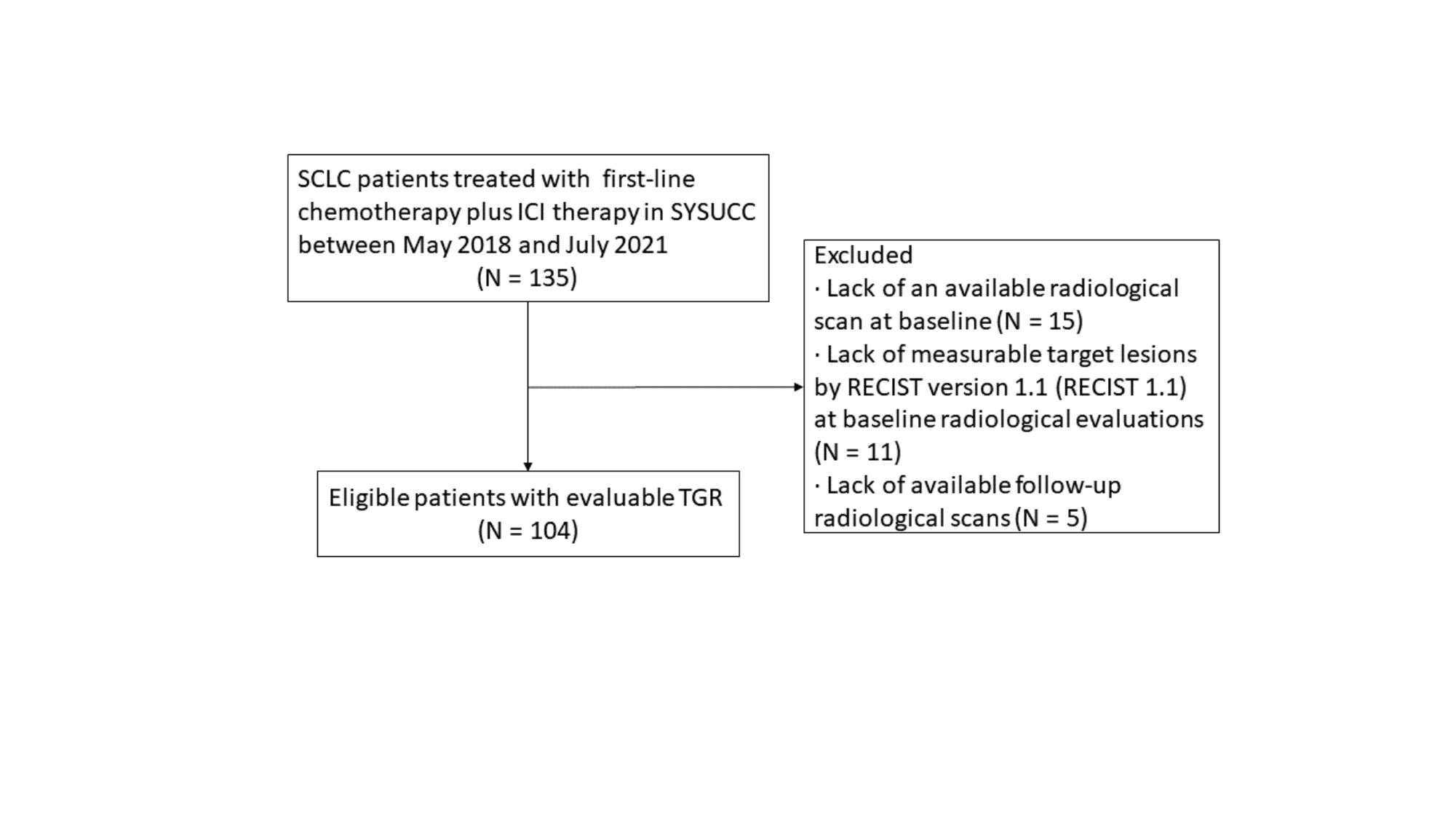

## Slide 2
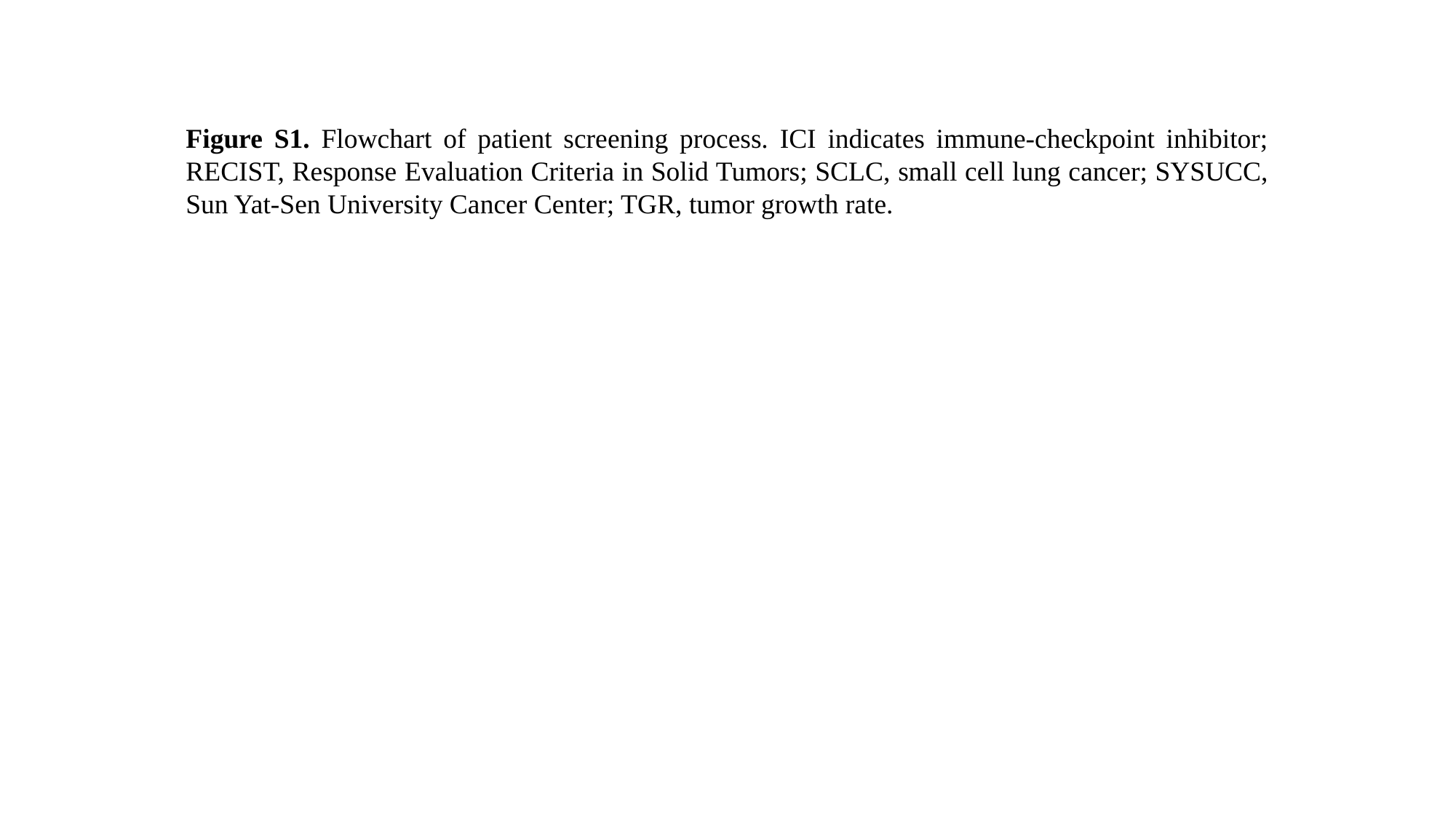

Figure S1. Flowchart of patient screening process. ICI indicates immune-checkpoint inhibitor; RECIST, Response Evaluation Criteria in Solid Tumors; SCLC, small cell lung cancer; SYSUCC, Sun Yat-Sen University Cancer Center; TGR, tumor growth rate.
